# Anthropogenic Influence on Prevalence of 2 Amphibian Pathogens

**DOI:** 10.3201/eid1407.070602

**Published:** 2008-07

**Authors:** Valerie St-Amour, Wai M. Wong, Trenton W.J. Garner, David Lesbarrères

**Affiliations:** * Laurentian University, Sudbury, Ontario, Canada; † Institute of Zoology, London, UK

**Keywords:** Ranavirus, chytrid fungus, emergent infectious diseases, anthropogenic activities, disease prevalence, amphibians, green frogs, rana clamitans, letter

**To the Editor:** Although the relationship between the emergence of zoonotic diseases and human influenced landscapes is accepted (*1*–*3*), the relationship between human-influenced landscapes and wildlife disease is less so. Evidence does support correlations between human activities and environmental conditions affecting wildlife disease emergence (*2*,*3*). These studies assume relationships between component(s) of human habitat modification and the virulence of disease, and derive estimates of virulence from counts of the visibly diseased or those that have seroconverted (*3*). This explains only part of the host and pathogen dynamic; it seems reasonable to extend the relationship to include prevalence of infection. Data supporting this extension are lacking. Here we present data from a study examining the correlations between human influences on habitat and prevalence of 2 amphibian pathogens (*Batrachochytrium dendrobatidis* and ranavirus FV3) in populations of *Rana clamitans* in central and northeastern Ontario, Canada.

We sampled an average of 25 animals (standard deviation ± 6.16) from 11 populations during summer 2005. We washed equipment in bleach and air-dried equipment between visits and sites. All animals were kept individually to avoid cross-contamination, euthanized with MS22, and assessed for infection using molecular diagnostics. We tested for ranavirus infection of livers by amplifying the major capsid protein using standard PCR (*4*). We tested for infection with *B. dendrobatidis* by using a quantitative real-time PCR (*5*). Prevalence for each pathogen was estimated as the proportion of animals testing positive at a pond.

Site coordinates were determined by using global positioning satellite (GPS), and 4 quantitative measures of human habitat modification were also assessed. GPS coordinates were used to map sites and to measure distance to the nearest road, industrial activity (agriculture, mine, paper mill), and human habitation; all measurements were in meters. We further assigned a qualitative measure of human influence on each breeding pond by assigning ponds to each of the following categories: 1) human presence without human habitat modification or extensive disturbance; 2) recreational activities (fishing, boating); 3) property development (housing or commercial buildings); 4) agricultural activity; and 5) industrial activity. Each of the 5 categories was assigned a 0/1 score; scores for each pond were summed 1–5 (by definition no site scored 0 due to sampling strategy) to derive the final measure of human influence. We modeled the relationship between prevalence and human habitat modification or influence using general linear models (GLM) with prevalence as the dependent variable and with all human influence variables log-transformed to meet assumptions of normality. A type III model structure was used to account for the influence of all explanatory variables in each analysis.

Eight ponds exhibited signs of FV3 infection (range 0%–63% prevalence); 6 ponds contained frogs infected with the amphibian chytrid (range 0%–36% prevalence). GLM did not show any relationship between the prevalence of chytrid infection and all of our explanatory variables (Table). In contrast, 3 of our explanatory variables had a significant influence of ranavirus prevalence. Distance to industrial activity (p<0.05), to human habitation (p<0.05), and degree of human influence (p<0.01) all had a significant effect on the dependent variable (Table).

**Table:** General linear models for the relationships of amphibian emerging infectious disease prevalence and anthropogenic variables

Data point	Degrees of freedom	Ranavirus			*Batrachochytrium dendrobatidis*	
		Mean squares	F value		MS	F value
Intercept	1	0.06	8.47 (p<0.05)		0.05	4.61 (p<0.1)
Human disturbance	1	0.24	35.35 (p<0.01)		0.0009	0.08
Distance to road	1	0.03	4.11 (p<0.1)		0.03	2.82
Distance to industry	1	0.06	8.82 (p<0.05)		0.07	5.89 (p<0.1)
Distance to housing	1	0.06	8.08 (p<0.05)		0.06	4.87 (p<0.1)
Error	5	0.01			0.01	

The disparity between results for the 2 pathogens generates several possible hypotheses. First, proximity to human activities may correlate with the probability of pathogen introduction through introduced species (*6*), fomites, or other sources of infectious particles, with the likelihood of introduction higher for ranavirus. Certainly, both pathogens are presumed to be vectored in association with human activities (*7*,*8*), but *B. dendrobatidis* exhibits a greater host and geographic range and thus should exhibit greater prevalence if humans were mediating introduction across the range of our study. Second, human activities such as construction and industry, may directly or indirectly influence the basic reproductive number, *R*_0_, of ranavirus to a greater extent than for that of *B. dendrobatidis*. Although ranavirus does exhibit optimal environmental ranges for replication and infection, the virulence of *B. dendrobatidis* can be directly influenced by the environment (*2*). Furthermore, infection by *B. dendrobatidis* occurs through a free-living stage; ranavirus is more likely transmitted through direct contact, which suggests that *B. dendrobatidis* would be more sensitive to environmental factors. Last, human activities may influence host ability to mediate immune responses that have the capability to prevent infection. Evidence exists that amphibian host responses to ranavirus are predominantly acquired (*9*); those for *B. dendrobatidis* may be more innate and less prone to environmental manipulation (*10*). Although the observed correlation should be further tested and the disturbance index should be refined, we believe our observed pattern may reflect the influence of human activities and habitat modification in the dispersal of infectious diseases. With increasing evidence pointing towards the role of emerging infectious diseases in the decline of amphibian populations, management plans should therefore account for the indirect effects related to human activities.

## References

[1] Jones KE, Patel NG, Levy MA, Storeygard A, Balk D, Gittleman JL, Global trends in emerging infectious diseases. Nature. In press. 10.1038/nature06536 PMC5960580 18288193

[2] Bosch J, Carrascal LM, Durán L, Walker S, Fisher MC. Climate change and outbreaks of amphibian chytridiomycosis in a montane area of Central Spain; is there a link? Proc Biol Sci. 2007;274:253–60. 10.1098/rspb.2006.3713 17148254 PMC1685858

[3] Bradley CA, Altizer S. Urbanization and the ecology of wildlife diseases. Trends Ecol Evol. 2007;22:95–102. 10.1016/j.tree.2006.11.001 17113678 PMC7114918

[4] St.-Amour V, Lesbarrères D. Genetic evidence of *Ranavirus* in toe clips: an alternative to lethal sampling methods. Conserv Genet. 2007;8:1247–50. 10.1007/s10592-006-9242-6

[5] Garner TWJ, Perkins M, Govindarajulu P, Seglie D, Walker SJ, Cunningham AA, The emerging amphibian pathogen *Batrachochytrium dendrobatidis* globally infects introduced populations of the North American bullfrog, *Rana catesbeiana.* Biol Lett. 2006;2:455–9. 10.1098/rsbl.2006.0494 17148429 PMC1686185

[6] Garner TWJ, Walker S, Bosch J, Hyatt A, Cunningham A, Fisher MC. Chytrid fungus in Europe. Emerg Infect Dis. 2005;11:1639–41. 16355506 10.3201/eid1110.050109 PMC3366739

[7] Jancovich JK, Davidson EW, Parameswaran N, Mao J, Chinchar G, Collins JP, Evidence for emergence of an amphibian iridoviral disease because of human-enhanced spread. Mol Ecol. 2005;14:213–24. 10.1111/j.1365-294X.2004.02387.x 15643965

[8] Fisher MC, Garner TWJ. The relationship between the introduction of *Batrachochytrium dendrobatidis*, the international trade in amphibians and introduced amphibian species. Fungal Biol Rev. 2007;21:2–9. 10.1016/j.fbr.2007.02.002

[9] Robert J, Morales H, Buck W, Cohen N, Marr S, Gantress J. Adaptive immunity and histopathology in frog virus 3-infected *Xenopus.* Virology. 2005;332:667–75. 10.1016/j.virol.2004.12.012 15680432

[10] Woodhams DC, Ardipradja K, Alford RA, Marantelli G, Reinert LK, Rollins-Smith LA. Resistance to chytridiomycosis varies among amphibian species and is correlated with skin peptide defenses. Anim Conserv. 2007;10:409–17. 10.1111/j.1469-1795.2007.00130.x

